# Clinical utility of rhythm control by electrical cardioversion to assess the association between self-reported symptoms and rhythm status in patients with persistent atrial fibrillation

**DOI:** 10.1016/j.ijcha.2021.100870

**Published:** 2021-09-15

**Authors:** Astrid N.L. Hermans, Nikki A.H.A. Pluymaekers, Theo A.R. Lankveld, Manouk J.W. van Mourik, Stef Zeemering, Trang Dinh, Dennis W. den Uijl, Justin G.L.M. Luermans, Kevin Vernooy, Harry J.G.M. Crijns, Ulrich Schotten, Dominik Linz

**Affiliations:** a Department of Cardiology, Maastricht University Medical Center and Cardiovascular Research Institute Maastricht, Maastricht, the Netherlands; b Department of Physiology, Maastricht University and Cardiovascular Research Institute Maastricht, Maastricht, the Netherlands; c Department of Cardiology, Radboud University Medical Center, Nijmegen, the Netherlands; d Center for Heart Rhythm Disorders, University of Adelaide and Royal Adelaide Hospital, Adelaide, Australia; e Department of Biomedical Sciences, Faculty of Health and Medical Sciences, University of Copenhagen, Copenhagen, Denmark

**Keywords:** Atrial fibrillation, Electrical cardioversion, Symptom-rhythm correlation

## Abstract

**Background:**

The best strategy to assess the association between symptoms and rhythm status (symptom-rhythm correlation) in patients with atrial fibrillation (AF) remains unclear. We aimed to determine the clinical utility of rhythm control by electrical cardioversion (ECV) to assess symptom-rhythm correlation in patients with persistent AF.

**Methods:**

We used ECV to examine symptom-rhythm correlation in 81 persistent AF patients. According to current clinical practice, the presence of self-reported symptoms before ECV and at the first outpatient clinic follow-up visit (within 1-month) was assessed to determine the prevalence of a symptom-rhythm correlation (defined as self-reported symptoms present during AF and absent in sinus rhythm or absent in AF and yet relief during sinus rhythm). In addition, we evaluated symptom patterns around ECV.

**Results:**

Only in 18 patients (22%), a symptom-rhythm correlation could be documented. Twenty-eight patients (35%) did not show any symptom-rhythm correlation and 35 patients (43%) had an unevaluable symptom-rhythm correlation as these patients were in symptomatic AF both at baseline and at the first outpatient AF clinic follow-up visit. Importantly, self-reported symptom patterns around ECV were intra-individually variable in 10 patients (12%) without symptom-rhythm correlation (of which 9 patients (11%) had AF recurrence) and in 2 patients (2%) with an unevaluable symptom-rhythm correlation.

**Conclusions:**

In patients with persistent AF, symptom assessment around rhythm control by ECV, once before ECV and once within 1-month follow-up, rarely identifies a symptom-rhythm correlation and often suggests changes in symptom pattern. Better strategies are needed to assess symptom-rhythm correlation in patients with persistent AF.

## 1 Introduction

Patient-tailored management of persistent atrial fibrillation (AF) relies on rate and/or rhythm control, antithrombotic treatment and management of concomitant cardiac diseases [1]. One of the main goals of AF rhythm control is amelioration of symptoms. Although a large proportion of patients with AF reports symptoms [2], it often remains unclear whether all symptoms are related to AF or whether also other concomitant cardiovascular or non-cardiovascular conditions and risk factors contribute to overall symptom burden in an individual patient. Knowledge about the association between symptoms and rhythm status (symptom-rhythm correlation) has potential clinical implications as it may identify patients who profit from rhythm control in regard to reduction in symptom burden and improvement in quality of life. However, standardized strategies to assess symptom-rhythm correlation are currently not available [3].

Electrical cardioversion (ECV) offers the opportunity to probe symptom-rhythm correlation. In patients in whom ECV is successful the time in sinus rhythm can be used to evaluate whether symptoms improve once sinus rhythm is restored (symptom-rhythm correlation), or whether symptom burden remains unaffected (no symptom-rhythm correlation) [4].

In this retrospective observational cohort study, we determined the clinical utility of rhythm control by ECV to assess symptom-rhythm correlation in patients with persistent AF. Therefore, in accordance with current clinical practice, we used self-reported symptom reports collected during the outpatient AF clinic visits before and after ECV to (1) examine the prevalence of a symptom-rhythm correlation (defined as self-reported symptoms present during AF and absent in sinus rhythm or absent in AF and yet relief during sinus rhythm), and (2) assess the symptom patterns around ECV in patients with persistent AF.

## 2 Methods

### 2.1 Study design

This retrospective observational cohort study complies with the Declaration of Helsinki and was approved by the Institutional Review Board at the medical center (Committee reference number: NL 45118.068.13). Staff members of the independent Clinical Trial Center Maastricht performed the study monitoring and data management. All patients provided written informed consent.

### 2.2 Study population

Hemodynamic stable patients with persistent AF who underwent ECV in Maastricht University Medical Center (Maastricht, The Netherlands) were included in this study. Individuals were excluded if they were aged <18 years, were on antiarrhythmic drugs, previously underwent ablation therapy for AF or if the current episode of AF was classified as postoperative AF. Other exclusion criteria were the presence of a pacemaker unable to detect AF with a regular paced rhythm during AF, and a history of myocardial infarction within four weeks preceding recruitment into the study.

### 2.3 Data collection

Baseline clinical characteristics (demographics, concomitant cardiovascular conditions, and medication) were retrieved from patient medical records. Furthermore, we obtained the presence of self-reported symptoms and the predominant self-reported symptom type (symptom with highest self-reported symptom burden) of each individual patient before ECV and at the first outpatient AF clinic follow-up visit (within one month after ECV) from patient medical records. During structured history taking, the presence of the following symptoms and their symptom-specific burden before and after ECV were interrogated by the attending physician without using a validated tool: palpitations, dyspnea, reduced exercise tolerance, tiredness, chest pain, and others. The presence of self-reported symptoms was determined to examine the prevalence of a symptom-rhythm correlation. Symptom-rhythm correlation was assessed by considering the association between self-reported symptoms and the rhythm status before and after ECV. Patients with symptoms prior to ECV and without symptoms in sinus rhythm as well as asymptomatic patients before ECV with yet symptom relief during sinus rhythm were defined as symptom-rhythm correlation. In persistent AF patients who perceived themselves as asymptomatic before ECV, ECV was performed to see if restoration and maintenance of sinus rhythm can ‘unmask’ a previously suppressed level of symptoms. The symptom-rhythm correlation was absent in patients with symptoms before ECV who remained symptomatic during sinus rhythm (regardless of changes in predominant symptom type) or in patients with symptoms prior to ECV and without symptoms in AF after ECV. Asymptomatic patients before ECV with or without symptoms in AF or sinus rhythm afterwards had no symptom-rhythm correlation as well. The symptom-rhythm correlation was unevaluable in patients who were symptomatic in AF before ECV and at the first outpatient AF clinic follow-up visit.

The predominant self-reported symptoms before and after ECV were collected to assess the symptom patterns around ECV. Intra-individually variable symptom patterns were defined as changes in predominant self-reported symptoms within patients around ECV.

### 2.4 Statistical analysis

All statistical analyses were performed using IBM SPSS 25.0 software (SPSS, Inc., Chicago, USA) and statistical significance was assumed at a 5% level. Histograms and Shapiro-Wilk tests were used to check for normality. Categorical variables were represented as numbers of patients (n) with percentages. Normally distributed continuous variables were reported as mean ± standard deviation (SD) and non-normal distributed continuous variables were presented as median with interquartile range (IQR). For the comparison of categorical data, the Pearson’s chi-squared tests or alternatively Fisher’s exact tests were used, as appropriate. Differences in continuous parameters were compared using one-way ANOVA and Kruskal-Wallis.

## 3 Results

### 3.1 Patients

A total of 81 patients were included in this analysis. The median age was 70 years (IQR, 64–75) and 19 patients (23%) were female. There were 51 patients (63%) with a first documented episode of AF and in 38 patients (47%) the current AF episode duration was ≤3 months (Table 1). Of all 81 persistent AF patients who underwent ECV, 63 were symptomatic (78%). ECV was performed in 18 additional persistent AF patients (22%) who perceived themselves as asymptomatic before ECV to see if restoration and maintenance of sinus rhythm can ‘unmask’ a previously suppressed level of symptoms. ECV was successful in 76 patients (94%), unsuccessful in 3 patients (4%), and 2 patients (2%) had immediate recurrence of AF (IRAF). Within one month after ECV, 52 patients (64%) had a documented recurrence of AF.

**Table 1:** Baseline characteristics of the patients with, without and with unevaluable symptom-rhythm correlation.

		Symptom-rhythm correlation			
	Total	Yes	No	Unevaluable	P-value
	(n = 81)	(n = 18)	(n = 28)	(n = 35)	
*Demographics*					
Female	19 (23)	7 (39)	8 (29)	4 (11)	0.06
Age (years), median (IQR)	70 (64–75)	69 (61–76)	71 (64–75)	70 (67–75)	0.71
Body mass index (kg/m^2^), mean ± SD, (n = 80)^b^	29.2 ± 4.6	28.5 ± 5.7	29.4 ± 3.9	29.4 ± 4.7	0.74
First detected atrial fibrillation^b^	51/77 (66)	10/18 (56)	19/27 (70)	22/32 (69)	0.55
Duration current atrial fibrillation episode ≤ 3 months^b^	38/79 (48)	8/18 (44)	12/27 (44)	18/34 (53)	0.76
Previous electrical cardioversion	13 (16)	4 (22)	3 (11)	6 (17)	0.59
Previous antiarrhythmic medication	5 (6)	2 (11)	0 (0)	3 (9)	0.22

*Concomitant cardiovascular conditions*					
CHA_2_DS_2-_VASc score ≥ 2^c^	65 (80)	13 (72)	23 (82)	29 (83)	0.62
Arterial hypertension	48 (59)	11 (61)	20 (71)	17 (49)	0.18
Stroke	7 (9)	1 (6)	3 (11)	3 (9)	1.00
Transient ischemic attack	8 (10)	0 (0)	2 (7)	6 (17)	0.15
Heart failure^b^^,^^d^	17/75 (23)	5/17 (29)	6/25 (24)	6/33 (18)	0.66
Obstructive sleep apnea syndrome	9 (11)	1 (6)	4 (14)	4 (11)	0.82
*Medication*					
Renin-angiotensin antagonists	42 (52)	12 (67)	14 (50)	16 (46)	0.34
Aldosterone antagonists	5 (6)	0 (0)	3 (11)	2 (6)	0.36
Anticoagulants	81 (100)	18 (100)	28 (100)	35 (100)	
Antiplatelets	5 (6)	2 (11)	1 (4)	2 (6)	0.71
Beta-blockers	69 (85)	17 (94)	26 (93)	26 (74)	0.07
Calcium channel blockers	17 (21)	3 (17)	7 (25)	7 (20)	0.78
*Dihydropyridine*^b^	13/17 (76)	2/3 (67)	7/7 (100)	4/7 (57)	0.18
Diuretics	31 (38)	7 (39)	10 (36)	14 (40)	0.94

Percentages may not total 100 because of rounding.

Values depicted as number of patients (n) with percentages unless indicated otherwise.

SD, standard deviation; IQR, interquartile range.

b Number of patients with available information is given since some patients had missing values.

c The CHA_2_DS_2_-VASc score is a well-established tool used for risk stratification of stroke in patients with atrial fibrillation, with scores ranging from 0 to 9 and a higher score corresponds to a greater risk. Congestive heart failure, hypertension, diabetes, vascular disease, an age of 65 years to 74 years and female gender are each allocated one point, and an age of more than 75 years and previous stroke or transient ischemic attack are each allocated two points [1].

d Heart failure was defined as a left ventricular ejection fraction of less than 40%.

### 3.2 Symptom-rhythm correlation

**Fig. 1:**
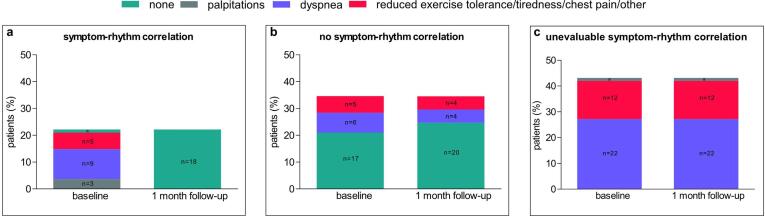
Symptom-rhythm correlation between baseline and 1-month follow-up in patients who underwent electrical cardioversion. Panel a shows details regarding the variability in symptom pattern between baseline and one month follow-up of patients with a symptom-rhythm correlation (n = 18). Panel b shows details regarding the variability in symptom pattern between baseline and one month follow-up of patients without a symptom-rhythm correlation (n = 28). Panel c shows details regarding the variability in symptom pattern between baseline and one month follow-up of patients with an unevaluable symptom-rhythm correlation (n = 35). ^a^ n = 1.

**Fig. 2:**
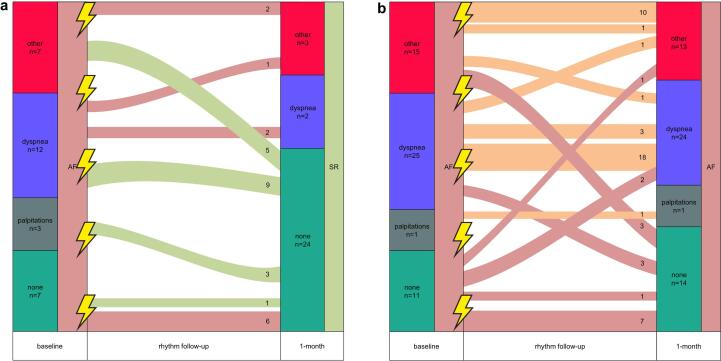
Symptom variability around electrical cardioversion per patient. Shown is the symptom variability around electrical cardioversion (ECV) among patients without recurrence of atrial fibrillation (AF) (panel a) and with recurrence of AF (panel b). Other includes the following symptoms: reduced exercise tolerance, tiredness, chest pain, and others. The green lines indicate patients with a symptom-rhythm correlation around ECV (defined as self-reported symptoms present during AF and absent in sinus rhythm (SR) or absent in AF and yet relief during sinus rhythm). The red lines indicate patients without a symptom-rhythm correlation around ECV. The orange lines indicate patients with an unevaluable symptom-rhythm correlation around ECV. The lightning symbols are used to display the moment of ECV. (For interpretation of the references to colour in this figure legend, the reader is referred to the web version of this article.)

The minority of patients (18 patients, 22%) displayed a symptom-rhythm correlation of which 17 **(21**%) had symptoms prior to ECV and no symptoms in sinus rhythm and 1 (1%) was asymptomatic before ECV with yet symptom relief during sinus rhythm (in this patient, ECV ‘unmasked’ a previously suppressed level of symptoms) (Fig. 1*, panel a;* Fig. 2*, panel a*). Twenty-eight patients (35%) did not show any symptom-rhythm correlation (Fig. 1*, panel b;* Fig. 2*, panel a and b)* and 35 patients (43%) with relapse of AF had an unevaluable symptom-rhythm correlation as these patients were in symptomatic AF both at baseline and at the first outpatient clinic visit (Fig. 1*, panel c;* Fig. 2 *panel b*). Baseline clinical characteristics of patients with and without symptom-rhythm correlation and of patients with an unevaluable symptom-rhythm correlation are reported in Table 1. All patient characteristics were comparable. The findings hold true when we excluded patients with prior attempts of rhythm control (previous ECV or antiarrhythmic medication therapy) because of potential ‘treatment expectation bias‘ (supplementary material online, *Table S1*).

### 3.3 Predominant self-reported symptoms

**Fig. 3:**
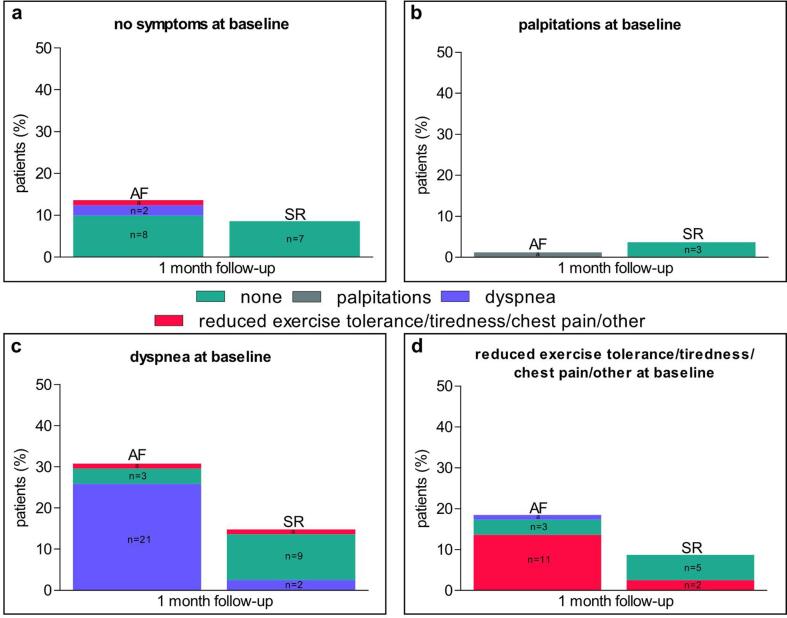
Symptom pattern before and after electrical cardioversion. Shown is the symptom pattern between symptoms at baseline and symptoms at one month follow-up among 81 patients with and without recurrence of atrial fibrillation (AF). Panel a-d shows the percentage of patients with a specified baseline symptom and their symptoms at 1-month follow-up. ^a^ n = 1. SR, sinus rhythm.

Before ECV, dyspnea was the most common symptom (n = 37, 46%), followed by reduced exercise tolerance (n = 16, 20%), tiredness (n = 5, 6%), palpitations (n = 4, 5%) and chest pain (n = 1, 1%). Twenty-two percent of patients (n = 18) reported no symptoms. Of the 29 patients with sinus rhythm after ECV, 24 (83%) were asymptomatic, 3 (10%) had reduced exercise tolerance and 2 (7%) had dyspnea at 1-month follow-up (Fig. 3*, panel a-d*). In the 52 patients with a recurrence of AF after ECV, there were 14 patients (27%) without symptoms, 24 (46%) with dyspnea, 8 (15%) with reduced exercise tolerance, 3 (6%) with tiredness, 1 (2%) with palpitations, 1 (2%) with chest pain and 1 (2%) with other symptoms at one month (Fig. 3*, panel a-d*). Importantly, self-reported symptom patterns around ECV were intra-individually variable in 10 patients (12%) without symptom-rhythm correlation (of which 9 patients (11%) had AF recurrence) and in 2 patients (2%) with an unevaluable symptom-rhythm correlation (Fig. 2*, panel a and b).*

## 4 Discussion

In this retrospective cohort study, the minority of patients showed a symptom-rhythm correlation (defined as predominant self-reported symptoms present during AF and absent in sinus rhythm or absent in AF and relief during sinus rhythm) around ECV. We found a high variability in self-reported symptoms before and after ECV in patients with AF recurrence.

### 4.1 Symptom-rhythm correlation and symptom pattern around electrical cardioversion

The low prevalence of symptom-rhythm correlation and the high remaining symptom burden after rhythm control by ECV do not support prior work showing that the majority of patients who were symptomatic in AF before ablation became asymptomatic in sinus rhythm after ablation [5], [6]. Additionally, patients with a successful ablation had greater reduction in symptoms compared to patients with an unsuccessful ablation [7], [8]. An explanation might be a different symptom perception in patients with paroxysmal and persistent AF, however invasive interventions per se may also lead to alterations in perception of AF caused by a placebo effect [8], [9], [10]. Moreover, patients’ limited prior knowledge of AF, previous health experiences and interactions with health care providers may influence symptom perception as well [11]. In general, blinded sham-controlled studies may be needed to definitely rule out a placebo effect of rhythm-control, particularly if symptom-burden is one of the main outcome-measures.

The main goal of rhythm control strategies is amelioration of symptoms in AF patients. In regard to symptom control, the best responder to rhythm control (by pharmacological interventions, ECV or AF-ablation procedures) would be an AF patient who is predominantly symptomatic because of AF-related symptoms. Furthermore, severe symptomatic patients would have a higher likelihood of symptom improvement after the achievement of sinus rhythm compared to minimally symptomatic or asymptomatic patients [10]. Therefore, the assessment of the underlying pathophysiological condition mainly contributing to symptoms is important to guide the decision for rhythm versus rate control. Theoretically, in symptomatic patients without symptom-rhythm correlation, non-AF related factors such as cardiovascular or non-cardiovascular conditions and risk factors, which do not change after successful rhythm control, are likely contributing to overall symptom burden in an individual patient. However, in our study, the cardiovascular conditions and risk factors of patients with and without symptom-rhythm correlation as well as of patients with an unevaluable symptom-rhythm correlation were quite similar. Importantly, in addition to the amelioration of symptoms, recent studies also showed that rhythm control (AF ablation therapy and treatment with antiarrhythmic drugs) may also be associated with a reduction in cardiovascular outcomes, potentially even irrespective of improvement of symptoms and in asymptomatic patients [12], [13], [14]. Therefore, the role of systematic symptom-rhythm correlation assessment using ECV as a diagnostic tool to guide decision on rhythm control in patients with persistent AF needs to be investigated in future studies [10], [15].

The assessment of symptom-rhythm correlation has potential clinical implications as it may identify patients likely profiting from rhythm control strategies to improve their symptom burden and quality of life. However, identifying a symptom-rhythm correlation in AF patients is challenging. The best way to determine symptom-rhythm correlation remains unclear. The high recurrence rate of AF within the first month is significantly limiting the diagnostic utility of ECV at one month. To enhance the performance of symptom-rhythm correlation assessment, the period in sinus rhythm after ECV may be lengthened by specific patient selection (e.g. smaller left atrial size) [16] or using temporary amiodarone or flecainide, which, however as such may affect symptom burden [4]. Besides, as it is established that ECV is associated with a 24-hour relapse gap of AF recurrence [17], symptom assessment at 24 h may give sufficient opportunity for an effective evaluation of changes in symptoms around ECV. Additionally, symptom burden was interrogated once at baseline and once at one month follow-up after ECV (in accordance with current clinical practice). A more longitudinal assessment of symptoms during simultaneous rhythm monitoring in persistent AF patients undergoing ECV may provide a more accurate approach to assess a symptom-rhythm correlation and to distinguish between AF-related symptoms (AF-symptoms) and unspecific disease-related symptoms (symptoms in AF). A better characterization and a better understanding of the mechanisms of symptoms in AF patients and symptom burden may help to obtain the correct diagnosis, chose an appropriate treatment (rhythm control vs. rate control), and assess the actual result of a treatment. Additionally, the absence of a clear symptom-rhythm correlation may provide a plausible basis for a structured assessment and then for targeted and comprehensive management of co-morbidities contributing to symptom burden.

There was a high variability in self-reported symptoms before and after ECV in patients with AF recurrence. This heterogeneity in terms of symptom presentation suggests that symptoms in patients with AF may be the manifestation of multiple pathophysiologic mechanistic pathways [3]. Patients with first-detected AF are more symptomatic than patients with a longer history of AF [18] and even in highly symptomatic AF patients, asymptomatic episodes may occur [9]. Moreover, there are higher rates of atypical symptoms in elderly with AF [19]. Although most AF patients experience symptoms during AF episodes [2], [20], symptom perception is highly variable [1], [9]. Sociodemographic- and sex-specific factors as well as anxiety- and depression-related mechanisms may be involved in the type or severity of self-reported symptoms in AF patients [21]. Additionally, symptoms in AF patients related to certain comorbidities such as heart failure, obesity, diabetes, coronary artery disease, arterial stiffness, and sleep-disordered breathing may perpetuate and contribute significantly to the perception and judgement of the frequency and severity of AF-related symptoms as well [22], [23], [24], [25]. Therefore, additional studies evaluating the effect of specific concomitant non-cardiovascular and cardiovascular conditions and risk factors on overall symptom burden are needed.

### 4.2 Limitations

Several limitations of our study should be mentioned. First, the sample size of our study was relatively small and there may be selection bias, as we included only those patients who were not on antiarrhythmic drugs. Therefore, there should be caution in generalizing our findings to all patients with persistent AF, as results may differ in other patient populations. Second, the presence of symptoms and if present, the predominant self-reported symptoms around ECV were obtained retrospectively from patient medical records (in accordance with current clinical practice). Thus, there is a risk that the coverage of different symptoms is not as complete as in a questionnaire, diary or structured interview. Third, we just applied one technique to assess symptom-rhythm correlation, namely assessment of symptoms once before ECV and once at one month after ECV (spot-check symptom assessment). A more longitudinal assessment of symptoms during simultaneous rhythm monitoring around ECV may provide a more accurate approach to assess a symptom-rhythm correlation. Further studies are required to test the utility of such approach. Fourth, we presented symptom-rhythm correlation as a categorical variable (yes or no). But probably, symptom-rhythm correlation assessment is not that “black or white”, as also other concomitant cardiovascular or non-cardiovascular conditions and risk factors may contribute to overall symptom burden. A point to take also into account is that prior work suggested that the physician’s assessment of AF-specific symptoms is an underestimation of patients AF-specific symptoms, especially when they are mild, which may affect the variability in symptoms and thus the prevalence of symptom-rhythm correlation around ECV [26].

## 5 Conclusions

In patients with persistent AF, spot-check-based symptom-rhythm correlation assessment around rhythm control by ECV, once before ECV and once at the first outpatient AF clinic follow-up visit (within one month after ECV), rarely identifies a symptom-rhythm correlation. Additionally, ECV often suggests changes in symptom pattern. Further research is warranted to identify more optimal strategies to assess symptom-rhythm correlation in patients with persistent AF and to establish the clinical implications of symptom-rhythm correlation assessment for AF management.

## Declaration of Competing Interest

The authors declare that they have no known competing financial interests or personal relationships that could have appeared to influence the work reported in this paper.
