# Supplementary material for: Clinical utility of rhythm control by electrical cardioversion to assess the association between self-reported symptoms and rhythm status in patients with persistent atrial fibrillation
Source: Int J Cardiol Heart Vasc. 2021 Sep 15;36:100870. doi: 10.1016/j.ijcha.2021.100870 (PMC8449169; doi:10.1016/j.ijcha.2021.100870)
Supplement: Supplementary Data 1 [file mmc1.docx]

**Supplementary material online**

**Table S1. Baseline characteristics of the patients without prior attempts of rhythm control.**

|  | Total  (n=65) | Symptom-rhythm correlation  Yes No Unevaluable P-value  (n=13) (n=25) (n=27) | | | |
| --- | --- | --- | --- | --- | --- |
| *Demographics* |  |  |  |  |  |
| Female | 15 (23) | 5 (39) | 7 (28) | 3 (11) | 0.12 |
| Age (years), median (IQR) | 70 (64-75) | 69 (59-75) | 70 (64-75) | 70 (66-74) | 0.77 |
| Body mass index (kg/m²), median (IQR) | 29.05 (25.10-32.15) | 29.03 (23.04-34.87) | 29.35 (26.72-31.77) | 28.68 (24.93-32.11) | 0.85 |
| First detected atrial fibrillation ^b^ | 50/62 (81) | 9/13 (69) | 19/24 (79) | 22/25 (88) | 0.35 |
| Duration current atrial fibrillation episode ≤3 months ^b^ | 31/63 (49) | 6/13 (46) | 11/24 (46) | 14/26 (54) | 0.83 |
| *Concomitant cardiovascular conditions* |  |  |  |  |  |
| CHA_2_DS_2-_VASc score ≥2 ^c^ | 52 (80) | 9 (69) | 20 (80) | 23 (85) | 0.50 |
| Arterial hypertension | 40 (62) | 8 (62) | 19 (76) | 13 (48) | 0.12 |
| Stroke | 6 (9) | 0 (0) | 3 (12) | 3 (11) | 0.64 |
| Transient ischemic attack | 6 (9) | 0 (0) | 1 (4) | 5 (19) | 0.13 |
| Heart failure ^b d^ | 15/61 (25) | 4/13 (31) | 5/22 (23) | 6/26 (23) | 0.84 |
| Obstructive sleep apnea syndrome | 7 (11) | 1 (8) | 4 (16) | 2 (7) | 0.59 |
| *Medication* |  |  |  |  |  |
| Renin-angiotensin antagonists | 34 (52) | 9 (69) | 12 (48) | 13 (48) | 0.39 |
| Aldosterone antagonists | 4 (6) | 0 (0) | 3 (12) | 1 (4) | 0.41 |
| Anticoagulants | 65 (100) | 13 (100) | 25 (100) | 27 (100) |  |
| Antiplatelets | 5 (8) | 2 (15) | 1 (4) | 2 (7) | 0.42 |
| Beta-blockers | 56 (86) | 13 (100) | 23 (92) | 20 (74) | 0.05 |
| Calcium channel blockers | 13 (20) | 1 (8) | 6 (24) | 6 (22) | 0.46 |
| *Dihydropyridine* ^b^ | 11/13 (85) | 1/1 (100) | 6/6 (100) | 4/6 (67) | 0.54 |
| Diuretics | 27 (42) | 5 (39) | 10 (40) | 12 (44) | 0.92 |

Percentages may not total 100 because of rounding. ^a^ Values depicted as number of patients (n) with percentages unless indicated otherwise. ^b^ Number of patients with available information is given since some patients had missing values. ^c^ The CHA_2_DS_2_-VASc score is a well-established tool used for risk stratification of stroke in patients with atrial fibrillation, with scores ranging from 0 to 9 and a higher score corresponds to a greater risk. Congestive heart failure, hypertension, diabetes, vascular disease, an age of 65 years to 74 years and female gender are each allocated one point, and an age of more than 75 years and previous stroke or transient ischemic attack are each allocated two points. ^1 d^ Heart failure was defined as a left ventricular ejection fraction of less than 40%. ^e^ IQR, interquartile range.
